# Limiting Dietary Lysine Increases Body Weight Variability by Restricting Growth Potential of the Lightest Growing Pigs

**DOI:** 10.3390/ani12040528

**Published:** 2022-02-21

**Authors:** Pau Aymerich, Carme Soldevila, Jordi Bonet, Josep Gasa, Jaume Coma, David Solà-Oriol

**Affiliations:** 1 Vall Companys Group, 25191 Lleida, Spain; csoldevila@vallcompanys.es (C.S.); jbonet@vallcompanys.es (J.B.); jcoma@vallcompanys.es (J.C.); 2 Animal Nutrition and Welfare Service, Department of Animal and Food Sciences, Universitat Autònoma de Barcelona, 08193 Barcelona, Spain; josep.gasa@uab.cat (J.G.); david.sola@uab.cat (D.S.-O.)

**Keywords:** body weight, variability, coefficient of variation, lysine, growing pig

## Abstract

**Simple Summary:**

Finding strategies to manage variability in growing pig operations is of major importance to avoid the extra costs associated with inefficient barn usage or penalties at the processing plants when pigs are not correctly sorted. The aim of this study was to determine whether dietary amino acid density can have an impact on body weight variability in a population of growing pigs. Feeding diets with a reduced amino acid density had a negative effect because they increased body weight variability of pens composed of the smallest pigs. For instance, in those pigs, the coefficient of variation, which is used as a measure of variability, increased significantly at the end of the experiment when dietary amino acid density was reduced. It was observed that this effect was explained by a growth restriction that was more severe the lightest the pigs were at the start of the trial. Hypothesizing that posteriorly there would be no differences in growth between those restricted pigs and the normally fed ones, the differences in growth would result in around 5–10 additional days to reach marketing weight. In conclusion, severe nutritional restrictions can negatively affect growing pigs’ body weight variability.

**Abstract:**

The goal of this experiment was to determine the implications of dietary standardized ileal digestible lysine (SID Lys:NE) on body weight variability of growing pigs grouped in three initial body weight categories (BWCAT). Animals (*N* = 1170) were individually weighed and classified in 3 BWCAT (Lp: 32.1 ± 2.8 kg, Mp: 27.5 ± 2.3 kg, and Sp: 23.4 ± 2.9 kg). Afterward, pens were randomly allocated to five dietary SID Lys:NE treatments (3.25 to 4.88 g/Mcal) that were fed over 47 days. Pen coefficient of variation of Sp at day 47 was linearly increased when reducing SID Lys:NE (*p* < 0.01), from 9.8% to 15.4% between the two extreme dietary levels. The linear effect was different in Sp compared to Lp (*p* < 0.05) as no effect of SID Lys:NE was reported in the latter BWCAT (*p* = 0.992). Further analysis showed that this effect was explained by a growth restriction that was more severe the lightest the pigs were at the start of the trial. To summarize, swine body weight variability can be negatively affected when SID Lys requirements are not fulfilled.

## 1. Introduction

Body weight (BW) variability is one of the major concerns in all-in-all-out swine production systems because of its detrimental effects on barn usage [1,2,3] and carcass uniformity [4]. In a population of growing pigs, variation in BW at the start of the growing period might result in differences of around 30–40 days to reach the same marketing BW [5]. This longer time entails a reduction in the number of batches per barn and year, representing relevant economic losses for the farmer and the system in general [1]. A major part of marketing BW variability is explained by birth BW [5,6]. However, some authors suggest that there might be room for improvement at later stages. For instance, nursery exit weight is considered a risk factor for slow growth [7], while other studies have proved the capacity of small pigs to catch up with their heavier counterparts during fattening [8]. The latter is clearly observed in studies showing how BW variability in absolute magnitude, usually expressed as the standard deviation (SD)s increases with the age of the pigs, whereas the coefficient of variation (CV) decreases [5,9]. Thus, showing that part of the variability is yet generated during the growing period.

Numerous strategies such as prolonging lactation [10], using complex diets at weaning [11], increasing amino acid density [12,13], energy density [14], or sorting by BW [15] have already been proved as useful in some conditions to increase BW of the lightest pigs. Nevertheless, the impact of some of those strategies, especially the nutritional ones, on BW variability still remains unclear. There are two main strategies to reduce BW variability, depending on whether the goal is to reduce the performance of the largest pigs or to improve the performance of the lightest ones. The first one can be easily achieved by restricting individual feed intake and has already been shown as a successful strategy, although with a relevant associated cost of implementation [16]. The second one could be theoretically achieved by giving greater nutrient-dense diets to pigs that might be fed under their requirements. For instance, in silico models have shown that increasing the number of feeding phases to better match individual nutrient requirements might be a strategy to reduce growth variability [17]. Although some studies have not reported coherent effects of both increasing starter diet allowances and providing a finishing diet with a greater energy and lysine density on BW variability [18], López-Vergé et al. observed a reduction in CV (9.7% vs. 11.3%) when small pigs were allowed the same amount of the starter diet as the largest ones at the beginning of the growing period [19]. Other authors have shown that although adding fat to increase dietary energy density increases the growth of the lightest pigs, it does not affect BW variability [14].

The hypothesis of the present study was that the greater effect of SID Lys:NE on BW gain of small pigs when grouped in BWCAT’s shown previously in Aymerich et al. [12] could be observed in body weight variability. Therefore, this study compared the response to SID Lys:NE between BWCAT’s in terms of body weight variability and individual BW gain.

## 2. Materials and Methods

The techniques used in this experiment were in accordance with the EU Directive 2010/63/EU for animal experiments. A more detailed explanation of the materials and methods, specifically feed ingredient composition and analyses, can be found in Aymerich et al. [12].

### 2.1. Experimental Design and Animals

In this trial, the divergent effect of SID Lys:NE on body weight variability between BWCAT’s was evaluated in a dose-response experiment. For 47 days, 1170 growing pigs ((Pietrain × (Landrace × Large white), half boars and half gilts) were fed one of the 5 equidistant dietary SID Lys:NE levels, varying from 3.25 to 4.88 g SID Lys/Mcal NE. Previously, pigs were grouped in non-mixed pens of 13 pigs and individually weighted and pre-classified in 3 initial BWCAT as large (Lp: 32.1 ± 2.8 kg), medium (Mp: 27.5 ± 2.3 kg), or small (Sp: 23.4 ± 2.9 kg). Pigs had an age difference equal or lower to 7 days and originated from the same sow farm. There were 6 replicates per combination of SID Lys:NE and BWCAT, 3 of each sex.

### 2.2. Feeding

Two extreme feeds were formulated with a constant net energy value (2460 kcal NE/kg), with the low SID Lys:NE being 3.25 and the high one 4.88 g SID Lys/Mcal NE. In both feeds, it was ensured that ideal protein ratios between essential amino acids (AAs) and lysine were satisfied [20]. Once in the farm, those feeds were mixed in 5 proportions to ensure equidistant SID Lys:NE levels using an automatic distribution feeding system.

### 2.3. Calculations and Statistical Analysis

Individual body weight was measured and used to calculate pen body weight SD. Subsequently, SD and pen BW were used to calculate the CV for days 0, 26, and 47. Statistical analyses were performed with R [21]. The nlme package was used to implement the models and the ANOVA [22]. Orthogonal polynomial contrasts, least-square means, and standard errors of the means (*SEM*) were carried out using the emmeans package [23]. The divergent response to SID Lys:NE between each pair of BWCAT’s on CV at days 0, 26, and 47 was studied in a linear model using SID Lys:NE, BWCAT, sex, and their interactions as fixed effects. Initial pen CV was added as a covariate in the models for days 26 and 47. Sex was finally removed from the model because it was not significant from the overall period, allowing a simpler model to be used. Different variance groups were empirically defined for each combination of SID Lys:NE and BWCAT in a weights statement to deal with heteroscedasticity based on residual plots as long as it resulted in an improvement in model fit [24].

Moreover, the hypothesis of a lower 1/3 and 2/3 quantile for individual weight in the limiting SID Lys:NE for each BWCAT and dietary treatment was tested with a two-sample rank test from the EnvStats package [25]. This analysis was carried out comparing the best performing SID Lys:NE (4.47 g/Mcal, T2), considered the control group, versus each of the other dietary treatments. Density plots of individual BW were obtained for each combination of BWCAT and SID Lys:NE, at days 0 and 47, using the ggridges package [26]. Finally, the linear relationship between individual body weight at the start and at the end of the trial, days 0 and 47, respectively, was compared in a linear model including the interaction between initial body weight, SID Lys:NE, sex, and their interactions as fixed effects. Pen was included as a random effect in the model to improve model fit considering the nested structure of the data [22]. A reference grid with different initial BW was used to determine the final BW for each SID Lys:NE using the emmeans package [23]. For all analyses, results were declared as significant or tendency when *p* ≤ 0.05 and 0.05 < *p* ≤ 0.10, respectively

## 3. Results

The Results section is further divided into three different analyses performed to evaluate the effects of SID Lys:NE on body weight variability in growing pigs. The first subsection includes analysis using pen as experimental unit and CV as the target variable. The other two subsections used the individual pig as an experimental unit, and the target variable is BW at the beginning and at the end of the experiment. Although not reported in this study, mortality and morbidity rates were not significantly affected by SID Lys:NE in a linear or quadratic manner, but a greater rate was observed in Sp than in Lp pens (3.5% vs. 0.5%, respectively).

### 3.1. Interaction between SID Lys:NE and BWCAT on Pen CV

Initially, a triple interaction model was fitted for all variables but was only significant in the initial model. Therefore, initial CV was used as a covariate to account for the differences observed in the initial distribution, and sex was finally excluded from the model for its absence of significance. As of then, it was no longer significant for CV on days 26 and 47. The effect of CV at day 0 on CV at day 26 and 47 was significant (*p* < 0.001), and therefore, it was included as a covariate, as the interaction with the other fixed effects was not significant (*p* > 0.050). The predicted CV at day 47 depending on SID Lys:NE without considering the BWCAT effect was 9.6%, 10.0%, 10.0%, 9.7%, and 11.8% for 4.88, 4.47, 4.07, 3.66, and 3.25 g SID Lys/Mcal NE, respectively.

The results from the interactive effects between SID Lys:NE and BWCAT on CV are reported in Table 1. At the start of the experiment, only Mp showed a linear effect of SID Lys:NE on the CV (*p* = 0.027), which was reduced at greater ratios. However, these differences were corrected by the inclusion of initial CV as a covariate in the models for days 26 and 47. At day 26, reducing SID Lys:NE increased CV of Sp in a linear manner (*p* < 0.001) but not in Mp or Lp (*p* ≥ 0.729). In addition, the linear response was significantly different between Sp and Mp (*p* = 0.017) and between Sp and Lp (*p* = 0.001). Similar results were reported at the end of the experiment, although then the differential linear effect was only significant between Lp and Sp (*p* = 0.017), whereas a tendency was reported between Sp and Mp (*p* = 0.064). The significant linear increase in CV while reducing SID Lys:NE in Sp (*p* = 0.004) was mainly the result of a greater CV at 3.25 g SID Lys/Mcal NE, with a CV of 15.4% ± 1.37% contrasting with a 9.8% ± 0.93% at 4.88 g SID Lys/Mcal NE. Pairwise comparison showed no significant differences between the final CV of the 15 different treatments, except for Sp fed 3.25 g SID Lys/Mcal NE that was significantly different from all the others except Sp fed 4.07 and 4.47 g SID Lys/Mcal NE.

### 3.2. Quantile Test for Initial and Final Individual BW

In this section, the results from the quartile test are presented to discern whether the effect observed in the previous section of a greater CV in the low SID Lys:NE in Sp could be observed taking the overall individual data distribution. Table 2 shows the 1/3 and 2/3 quantiles for each combination of SID Lys:NE and BWCAT for individual BW at days 0 and 47, at the beginning and at the end of the trial. The statistic test comparing the SID Lys:NE showing a greater quantile (4.47 g/Mcal) with the other ones showed that at day 0, there were no significant differences for both quantiles (*p ≥* 0.117). However, at day 47, the 1/3 quantile of 3.25 g SID Lys/Mcal was significantly lower in the three BWCAT, although the magnitude of the difference was greater in the Sp, with 5.3 kg less. Regarding the 2/3 quantile analysis, the results showed a similar reduction as in the 1/3 for Mp (*p* = 0.021). No significant differences were reported for Sp (*p* = 0.242) but found a tendency for a lower quantile for Lp (*p* = 0.095).

**Figure 1:**
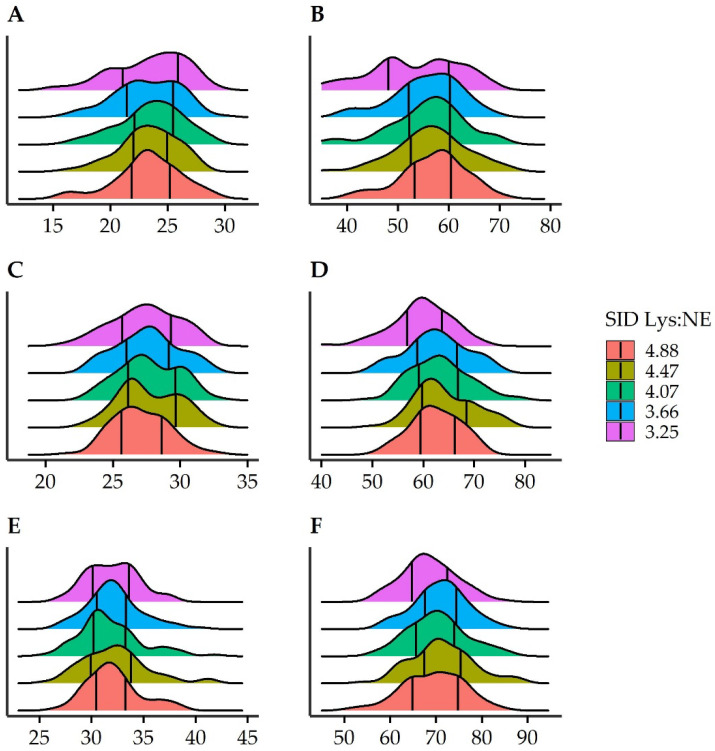
Density plots of initial and final individual body weight (BW) distribution by standardized ileal digestible lysine to net energy ratio (SID Lys:NE) for each body weight category (BWCAT). Pen BWCAT were small (**A**,**B**, 23.4 ± 2.9 kg), medium (**C**,**D**, 27.5 ± 2.3 kg) or large (**E**,**F**, 32.1 ± 2.8 kg). The plots on the left side are for BW at day 0, whereas the ones on the right side are of BW at day 47. Vertical lines represent the 1/3 and 2/3 quantiles.

To further illustrate the results from the quantile analyses, Figure 1 shows the density distribution by SID Lys:NE in each pen BWCAT at the beginning and at the end of the experiment. The plot shows that in Sp, the lowest SID Lys:NE resulted in a density distribution with two picks, which led to a severe reduction in the 1/3 quantile.

### 3.3. Relationship between Initial and Final Individual BW

**Figure 2:**
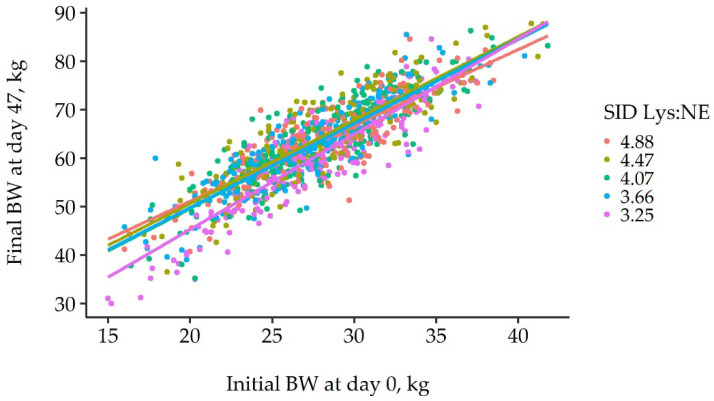
Scatterplot of individual initial and final body weight (BW), day 0 and 47, respectively, depending on the standardized ileal digestible lysine to net energy ratio (SID Lys:NE) fed. Lines represent the best fit of the multiple regression mixed model, including the interaction between initial BW and SID Lys:NE to predict final BW. The regression equations are: 19.8 + 1.57 × BW_d0_, 16.1+ 1.72 × BW_d0_, 14.5+ 1.76 × BW_d0_, 15.0 + 1.74 × BW_d0_, 5.9 + 1.97 × BW_d0_ for 4.88, 4.77, 4.07, 3.66 and 3.25 g SID Lys/Mcal NE.

The relationship between initial and final individual BW by SID Lys:NE was analyzed in a linear mixed model, initially including the effect of sex. However, as neither the main effect nor interactions were significant (*p* ≥ 0.109), those were finally removed from the model. Therefore, the final model predicted final BW using initial BW, SID Lys:NE, and their interactions as fixed effects (Figure 2). This model reported a significant interaction between initial BW and SID Lys:NE (*p* = 0.006), and, besides, both main effects were also significant (*p* < 0.001). The interaction was the result of a different slope for 3.25 compared to 4.88 g SID Lys/Mcal NE (*p* = 0.003) whereas only tendencies were found with 3.66 (*p* = 0.065), 4.07 (*p* = 0.094) and 4.47 g SID Lys/Mcal NE (*p* = 0.091). Between the four greatest SID Lys:NE, no differences in slopes were reported when those were contrasted (*p* ≥ 0.788). This greatest slope, together with a lower intercept in the low SID Lys:NE, was the result of a lower final BW in the growing pigs with a lighter initial BW but not in the largest ones. For instance, when applying the regression equations for each treatment, pigs with an initial BW of 20 kg would grow to 52.2 and 46.2 kg for the highest and lowest SID Lys:NE, respectively. However, when considering a pig with an initial BW of 35 kg, the predicted final BW for the same SID Lys:NE would be 76.5 and 76.7 kg, respectively. In statistical terms, considering 5 kg intervals in initial BW, there would be significant differences between treatments up to 30 kg, with a lower BW for pigs in the lowest SID Lys:NE compared to 4.47 or 4.88 g SID Lys/Mcal NE. Pigs with an initial BW equal or greater to 35 kg would not show significant differences in final BW with the SID Lys:NE used in this experiment.

## 4. Discussion

Variability will continue to be a relevant issue in all-in-all-out swine production systems because it is an inherent property of biological systems that will never be completely withdrawn [3,27]. However, all strategies that lead to a lower variability within the system might be considered both from an economic and efficiency perspective. In 2004, Patience et al. [1] calculated that reducing barn use by 2 weeks by growing period resulted in USD 1.25$ savings per pig due to lower space requirements. The magnitude of the reduced cost will depend on the difference of days required to reach the same marketing BW between the lightest and the heaviest pigs. This difference has been estimated to be around 30–40 days in commercial conditions, resulting from BW differences of around 15 kg at the start of the grow-finishing phase [5,28].

By way of example, the previously published productive results of the present study showed an ADG reduction of 80–90 g/d in the lowest SID Lys:NE for Sp, representing around 4 kg at the end of the experiment [12]. However, the multiple regressions for individual BW in this study reported a reduction of around 7 kg for pigs with an initial BW of 17.5 kg fed the lowest SID Lys:NE. Therefore, whereas when considering an average daily gain of 700 g/d Sp it would represent just 5.7 days more to reach the final BW of unrestricted pigs, those lightest would require at least 10 days more. Another way of illustrating such effect is by predicting the final BW for each SID Lys:NE considering initial BW of 20 and 35 kg with the multiple regression estimates. The prediction shows that the 15 kg between those pigs became 24.5 kg for 4.88 g SID Lys/Mcal NE, 30.4 kg for 3.25 g SID Lys/Mcal NE, and 26.2–26.4 for the remaining intermediate SID Lys:NE at day 47. Thus, comparing the two extreme SID Lys:NE, the days required to empty the fattening barn could be increased around 25%, which would represent at least one extra week.

The different analyses clearly indicate that the linear reduction in the CV of Sp was the result of a limitation when feeding 3.25 g SID Lys/Mcal NE than an advantage of feeding above the requirements. Only small numeric differences were observed between the four higher SID Lys:NE and no significant differences between BWCAT in the final CV were observed for the unrestricted pigs. The differences in final CV from 9.8% to 11.4% in the unrestricted treatments to 15.4% in the 3.25 g SID Lys/Mcal NE were consistent with the individual prediction of final BW. For instance, with an initial BW of 20 and 27 kg (average initial BW for Sp was 23.4 kg), in the lowest SID Lys:NE, those pigs’ final BW was around 7 and 4 kg lower than in the other SID Lys:NE, respectively. Therefore, the increase in SD would be around 1.5 kg, and therefore, the final CV with a BW of 52.7 kg would increase by around 3 % points. The authors acknowledge that in this type of trial, the perfect initial distribution of treatments would be preferred, but a suitable distribution of both BW and CV in a 15 treatment study including 2 sexes continues to be an important challenge. In this situation, the use of individual data for understanding BW variation is of great relevance.

Older studies did not detect a lower CV when feeding additional synthetic lysine, although higher BW was observed for those pigs [29]. On the contrary, López-Vergé et al. [19] found that a budget feeding strategy for the lightest growing pigs to ensure they were eating the required amount of the three first feeds increased the BW of those pigs 3.2 kg and reduced CV from 11.3% to 9.7%. Those effects are comparable to the ones obtained in our study, with an average reduction from 9.6% to 11.8% when pigs were fed a diet below their requirements. However, in that study, the nutritional restriction in SID Lys:NE for the ones fed by time and not by budget was probably lower than the study of López-Vergé et al. [19] considering the diets used. This might indicate that the positive effect that those authors found was related to giving the starter diet during the first two weeks in the fattening barn, starting to be fed at 18–19 kg for those small pigs. Contrarily, in the present experiment, the dietary treatments were applied when the small pigs had a BW of 23.4 kg, which might suggest a lower chance for improving the growth performance of the smallest pigs. This hypothesis is supported by evidence that the odds of a small pig not being light at marketing time diminish with the increasing age of the animal [5]. Furthermore, results might vary depending on the reason for the differences in BW between Sp and Lp. Future results should aim to distinguish whether the effect is just related to birth weight or to other variables such as lactation length, colostrum intake or post-weaning performance.

Another point of discussion is the loss of significance at day 47 of the differential linear effect of SID Lys:NE between BWCAT, mostly between Mp and Sp. Rather than a loss of the effect of SID Lys:NE on the CV of Sp, we would suggest that this was the result of a slight effect on the CV of Mp, although not significant, but greater than at day 26. Some effect on Mp could be expected, although lower than in Sp, considering that there were also some light pigs in those pens. Nevertheless, as this increase in CV was the result of feeding those pigs below their nutritional requirements for optimal performance, it could be hypothesized that part of the reduced BW might be overcome by compensatory growth. For this to happen, some conditions might be fulfilled, such as the ones described by Menegat et al. [30], and further experiments following pigs until marketing should be performed in the future. Finally, both quantile and multiple regression showed that the greater variability is related to a part of the smallest pigs, which are severely growth restricted. From the different figures, it could be established that this restriction was especially grave in pigs with an initial BW lower than 25 kg. This threshold meant that the restriction was principally effective on the 25% smallest pigs in the population. In addition, the density plots indicate that this effect resulted in two normal distributions within Sp fed the lowest SID Lys:NE, verifying the greater effect on the smallest pigs.

## 5. Conclusions

Results of this experiment indicate that severely limiting dietary SID Lys:NE below nutritional requirements for optimal performance can negatively affect the CV of a population of growing pigs (28–63 kg BW). This effect was the result of a growth restriction of the smallest pigs fed the lowest SID Lys:NE, but no positive effect was observed in the largest ones. Practical implications of this work would be avoiding limiting SID Lys:NE not only for the negative impact on productive parameters such as growth and feed efficiency but also for its detrimental effect on body weight variability.

## Figures and Tables

**Table 1:** Contrasting linear (Lin.) and quadratic (Quad.) effects of the standardized ileal digestible lysine to net energy ratio (SID Lys:NE) and pen initial body weight category (BWCAT) the coefficient of variation (CV) at days 0, 26, and 47 of the experiment.

Item	BWCAT ^3^	SID Lys:NE, g/Mcal					*p*-Value			
		3.25	3.66	4.07	4.47	4.88	Lin. ^1^	Quad. ^1^	Lin. ^2^	Quad. ^2^
CV d 0, %	Large	7.7	8.2	9.3	9.8	8.1	Lp/Mp *	Lp/Sp ^†^	0.225	0.173
	SEM	(0.49)	(0.90)	(1.56)	(0.90)	(0.49)				
	Medium	10.0	8.3	7.9	8.2	7.4			0.027	0.382
	SEM	(0.49)	(0.90)	(0.90)	(0.49)	(0.90)				
	Small	13.5	12.1	12.1	10.9	12.4			0.417	0.284
	SEM	(1.56)	(1.56)	(0.90)	(0.90)	(0.90)				
CV d 26, %	Large	9.8	9.8	9.3	10.1	9.8	Lp/Sp ** Mp/Sp *	-	0.852	0.662
	SEM	(0.46)	(0.45)	(0.44)	(0.44)	(0.70)				
	Medium	10.8	11.0	10.2	10.7	10.5			0.729	0.882
	SEM	(1.07)	(1.08)	(1.08)	(0.70)	(0.71)				
	Small	13.7	9.7	11.3	10.9	8.2			<0.001	0.807
	SEM	(0.74)	(0.47)	(1.08)	(0.70)	(0.72)				
CV d 47, %	Large	9.6	9.0	9.4	8.9	9.6	Lp/Sp * Mp/Sp ^†^	-	0.992	0.557
	SEM	(0.91)	(0.61)	(0.59)	(0.89)	(0.61)				
	Medium	10.5	10.2	9.1	10.1	9.3			0.290	0.657
	SEM	(0.89)	(0.61)	(0.66)	(0.61)	(0.63)				
	Small	15.4	10.0	11.4	11.0	9.8			0.004	0.135
	SEM	(1.37)	(0.92)	(1.34)	(0.90)	(0.93)				

Least square means. ^1^ Interaction between BWCAT for the orthogonal polynomial contrasts of SID Lys:NE effects: ^†^ 0.05 < *p* ≤ 0.10, * ≤0.05, ** ≤0.01. ^2^ Orthogonal polynomial linear contrasts of SID Lys:NE for each BWCAT. ^3^ BWCAT: large (32.1 kg), medium (27.5 kg) or small (23.4 kg). SEM: standard error of the mean.

**Table 2:** Individual body weight 1/3 and 2/3 quantile comparison by pen initial body weight category (BWCAT) and standardized ileal digestible lysine to net energy ratio (SID Lys:NE) at days 0 and 47 of the experiment.

BWCAT ^2^	SID Lys:NE, g/Mcal					*p*-Value Pairwaise vs. 4.47 ^1^			
	3.25	3.66	4.07	4.47	4.88	3.25	3.66	4.07	4.88
Large									
Day 0—1/3 quantile	30.8	31.2	30.4	30.9	31.0	0.567	0.914	0.307	0.696
Day 47—1/3 quantile	66.3	68.5	67.7	68.9	66.1	0.007	0.368	0.410	0.198
Day 0—2/3 quantile	33.2	32.9	32.6	33.3	32.8	0.500	0.433	0.250	0.250
Day 47—2/3 quantile	70.8	73.6	72.3	74.2	72.7	0.095	0.368	0.211	0.305
Medium									
Day 0—1/3 quantile	26.4	26.4	26.8	26.4	26.1	0.633	0.567	0.883	0.196
Day 47—1/3 quantile	58.2	59.9	60.4	61.4	60.2	<0.001	0.266	0.212	0.212
Day 0—2/3 quantile	28.5	28.4	28.9	28.9	28.1	0.198	0.198	0.567	0.117
Day 47—2/3 quantile	62.2	65.0	65.4	66.1	64.5	0.021	0.284	0.284	0.180
Small									
Day 0—1/3 quantile	22.5	22.1	22.6	22.4	22.7	0.633	0.368	0.802	0.750
Day 47—1/3 quantile	48.8	53.1	54.2	54.1	54.0	<0.001	0.525	0.592	0.569
Day 0—2/3 quantile	25.2	25.0	24.8	24.6	24.5	0.987	0.750	0.847	0.633
Day 47—2/3 quantile	57.8	59.5	59.0	59.6	59.6	0.242	0.478	0.477	0.430

^1^ Pairwise comparison of 4.47 g SID Lys/Mcal NE to the other SID Lys:NE treatments. ^2^ BWCAT: large (32.1 kg), medium (27.5 kg), or small (23.4 kg).

## Data Availability

The data presented in this study are available on request from the corresponding author.
